# Evidence for Clinical Use of Honey in Wound Healing as an Anti-bacterial, Anti-inflammatory Anti-oxidant and Anti-viral Agent: A Review

**DOI:** 10.17795/jjnpp-9487

**Published:** 2013-07-17

**Authors:** Reza Yaghoobi, Afshin Kazerouni, Ory kazerouni

**Affiliations:** 1 Department of Dermatology, Jundishapur University of Medical Sciences, Ahvaz, IR Iran; 2 Osteo Rehab Clinics, Brampton, Ontario, Canada

**Keywords:** Honey, Biological Dressings, Burns, Anti-inflammatory Agents

## Abstract

**Context:**

To provide an updated review of published literature on the anti-oxidant, anti-bacterial and anti-inflammatory properties of honey.

**Evidence Acquisition:**

CINAHL, BioMed Central, Cochrane Library, Medline and Embase data bases and reference lists were used to find randomized controlled trials and review articles. Randomized controlled trials using honey with a comparator were reviewed, along with published review articles to determine the relative benefits of tropical honey. These methods were undertaken by three reviewers.

**Results:**

Honey has anti-oxidant, anti-bacterial and anti-inflammatory properties. It can be used as a wound dressing to promote rapid and improved healing. These effects are due to honey’s anti-bacterial action, secondary to its high acidity, osmotic effect, anti-oxidant content and hydrogen peroxide content. The use of honey leads to improved wound healing in acute cases, pain relief in burn patients and decreased inflammatory response in such patients. However, it has proven to be ineffective in chronic leg ulcers. Overall, studies have been done in favor of the use of honey in medicine.

**Conclusions:**

Honey has almost equal or slightly superior effects when compared with conventional treatments for acute wounds and superficial partial thickness burns. More randomized controlled trials with significant statistical power comparing different kinds of honey, are required in order to create a strong body of evidence towards definite recommendations for medical use. There is biological plausibility.

## 1. Context

Honey is derived from nectar gathered and modified by the honeybee, *Apis mellifera*. It is a carbohydrate-rich syrup derived from floral and other plants nectars and secretions. Honey has been used in folk medicine since ancient times and has more recently been rediscovered by medical researchers for its use in dressing acute and chronic wounds. Traditionally, honey has been used to treat burns, infected and non-healing wounds and ulcers, boils, pilonidal sinus, venous and diabetic foot ulcers (1-6). Recent studies confirm the efficacy of honey in treating venous ulcers (7). In patients suffering from malignant wounds, improvement with respect to wound size and cleanliness was seen after treatment with honey-coated bandages (8). Similarly, honey dressing quickened rates of healing in pressure wounds. Honey has also been used to lessen foul odors emanating from wounds which cause a social barrier for patients and may lead to isolation. There are also reports of using medihoney to reduce the symptoms of chronic ocular surface diseases (9, 10). Unrefined honey has anti-inflammatory, anti-bacterial and anti-oxidant properties (11). The antibacterial action is due to its acidity, hydrogen peroxide content, osmotic effects, nutritional and antioxidant content, stimulation of immunity, and unidentified compounds (12). Different kinds of honey like Gelam, Medihoney, Tualang and Manuka, have been tested and found to have similar properties. Some forms of medically-certified honeys have been licensed as medical products for professional wound care in Europe and Australia (4, 10, 13). Medihoney exhibits a standard antibacterial activity, which has been confirmed by many *in vitro* testing studies. Medihoney is sterilized by gamma irradiation (10). As Clostridium botulinum spores exist in our environment (soil, air, dust and in agricultural products), it is imperative to inactivate the spores. If a deep wound is contaminated by bacteria in an anaerobic environment, there is the possibility that the proliferation of spores and production of botulinum toxin will occur. Botulinum toxin can have systemic effects such as paralysis and cardiac arrhythmia (10). Honey has been heated to eradicate the spores, and this takes place under 120 degrees centigrade temperature for 10 minutes. However, this can alter some of honeys’ beneficial properties. Glucose oxidase is not heat resistance. Therefore, gamma irradiation was introduced to destroy occasionally seen spores in honey, while having no adverse impacts on honey’s beneficial properties (10). Medihoney is not considered to be an antiseptic agent because it does not meet all criteria of a wound antiseptic. The following are four of them:

1) Quick effect on different kinds of bacteria and different fungi; 

2) Acceleration of the physiological process of wound healing (debridement and granulation); 

3) No local or systemic adverse effects (allergy or toxicity); 

4) Cost effectiveness. 

Honey meets all but the first criterion (10). A good body of evidence from published literature exists, recommending the routine clinical use of honey. Honey is the oldest wound dressing material known to human, when some modern products are failing in this area. Laboratory studies provide further evidence supporting its use in wound dressing due to its bioactivities. Honey stimulates leukocytes to release cytokines, which is what initiates the tissue repair process. It also stimulates immune response to infection. The stimulation of other aspects of the immune system by honey is also evident (Proliferation of B- and T- lymphocytes and the action of phagocytes). Honey stimulates the production of antibodies. It is suggested that this, is due to honey effect of enhancement of the immune system and antibacterial activity. The antibacterial activity of honey is in a broad spectrum as evidenced by many studies. The purpose of this review is to identify up-to-date evidence in order to bring additional armamentarium to a practitioners table for treating relevant conditions.

## 2. Evidence Acquisition

Three different reviewers searched medical databases (CINAHL, BioMed Central, Cochrane Library, Medline and Embase) for randomized controlled trials using honey with a comparator. They also reviewed published review articles to determine the relative benefits of tropical honey (keywords in combination were searched on different databases under title, abstract or all fields). Search was limited to English articles and those in the last 30 years among which there were RCTs (human and animals) and reviews. Authors were not contacted for original data. This was performed during the period of January 2012 to Dec 2012.

## 3. Results

### 3.1. Anti-bacterial Activity

Honey has been principally used for its antibacterial effects since ancient times (1). It was believed that honey could be used in the topical treatment of wounds and burns due to its anti-bacterial and wound healing promotion activity (2-4). Different mechanisms of action have been suggested for the anti-bacterial effects of honey. Its sugar content is high enough to hinder microbial growth. This is believed to be a result of its osmotic effect, which prevents the growth of bacteria and therefore promotes healing. The application of a topical sugar paste for the same purpose was also reported in many studies (4, 10, 11). The high sugar content of honey is not the sole reason for this effect. If honey is diluted with water to reduce its sugar content and osmotic effect, it is still able to inhibit the growth of many bacteria causing wound infection (10, 14-16). The antibacterial activity may be due to hydrogen peroxide activity, which is continuously produced by enzymes even when honey is diluted and remains well below the level that causes inflammatory effects (17). Some honeys additionally contain plant-derived antibacterial components: honey from some Leptospermum species has a very high level of such (17). For medical purpose honey needs to be sterilized by gamma-irradiation, which would not have any impact on antibacterial activity (10, 18). A review by Molan 1998 cites strong evidence supporting a shortened healing time for partial thickness burns and bed sores after using honey gauze in comparison with other dressings (19). The comparison was made between honey and polyurethane film (honey group healed in an average of 10.8 days and polyurethane group healed in 15.3 days), honey and amniotic membrane (honey group healed in 9.4 days and amniotic membrane group healed in 17.5 days), honey and boiled potato peel (honey group healed in 10.4 days and other group healed in 16.2 days), honey and silver sulfadiazine ( in honey group 87% healed in 15 days and second group only 10% healed in 15 days) and honey and saline (honey group healed in 8.2 and saline group healed in 9.9 days) (19). The results show a considerably shorter healing period when dressing the wound with a honey bandage (19). It is also noticed that using honey for dressing infected wounds gives it a clean, clear base that allows early grafting and an increased chance of acceptance. As a result, surgery would be more successful, especially in cases of wounds in diabetic patients (19). Researchers have failed to point out the active ingredient, while over 100 substances are candidate for antibacterial activity (10), Antibiotics attack the cell wall of bacteria in order to destroy it. Honey works in a different way. Honey is hygroscopic, meaning that it draws moisture out of the environment and dehydrates the bacteria with the aid of its hyperosmolar properties (honey is high in sugar) (14). It provides rapid autolytic debridement and wound deodorization (12, 15, 20). Honey has a mean pH of 4.4 (21, 22). The acidification of wounds speeds up healing and honey can also reduce wound colonization or infection as such conditions are often accompanied by a pH of > 7.3 in wound exudates (21-24).

### 3.2. Anti-inflammatory Properties

Aside from the fact that honey can remove bacteria that causes inflammation, a decrease in wound inflammation after applying honey gauze can be a result of honey’s direct anti-inflammatory properties (19). Even when there was no infection present, the anti-inflammatory effect was observed in animals. The anti-inflammatory effect has been observed by microscopic examination of wound tissues after applying honey on wounds in animal models (reduction in the number of white blood cells was observed) (19). Medihoney also benefits wound healing through its anti-inflammatory effects. The amount of wound exudates is due to the local inflammatory process around the wound. Therefore, the anti-inflammatory action of honey reduces edema and exudates, which can subsequently improve wound healing. This effect also reduces pain caused by pressure on nerve endings and reduces the amount of prostaglandin produced in inflammatory process (10). The anti-inflammatory effects of honey have been observed in animal models as well in clinical settings (14, 25, 26). The evidence from animal studies may be more convincing. Animals do not show placebo effect and are free from bias as they are incapable of having behavioral influences on the healing process (10). Honey’s anti-inflammatory action and stimulatory effects on granulation and epithelialisation, help in rapidly reducing pain and edema (12, 20). By providing moist healing, it can minimize hypertrophic scarring (12, 20). Honey also stimulates the angiogenesis, granulation and epithelialisation, which helps speed up the healing process (14, 27, 28). Honey can trigger the sequence of events to enhance angiogenesis and proliferation of fibroblasts and epithelial cells by producing certain growth factors like Tumor Necrosis Factor (TNF-alpha) (29). In fact, 5.8 kilodalton, a component of honey, can stimulate a response in macrophage which would trigger and accelerate the production of growth factors that affect epithelial cells and fibroblasts (10). Some compounds like prostaglandins and nitric oxide are major players in the process of inflammation. Honey is known to increase nitric oxide end products and decrease the prostaglandin levels (30). The acidification of wounds can enhance healing due to honey’s low pH. Honey’s low pH can enhance off loading oxygen from hemoglobin in capillaries. It can also suppress protease activity in wounds because of non-neutral pH which is not favorable for their activities (10). Increased protease activity in wounds can either slow or stop healing by destroying growth factors and the protein fibers and fibronectin in wounds, which is necessary for the activation of fibroblasts and migration of epithelial cells. This protease activity is a result of extra inflammatory reactions (10). The anti-inflammatory activity of honey can eliminate this obstacle to healing. The antibacterial activity of honey works by removing infectious bacteria stimulating the inflammatory response. Honey has debriding action which helps to reduce the sources of bacteria and hence prevent further inflammatory reactions (10).

### 3.3. Anti-oxidant Properties

Phytochemicals are responsible for the anti-oxidant activity of honey, and the anti-bacterial activity of honey is partly due to the presence of phytochemical components (28). Different antioxidants present in honey include flavonoids, monophenolics, polyphenolics and vitamin C (31-33). Free radicals derived from oxygen also known as Reactive Oxygen Species (ROS), are produced by the respiratory mitochondrial chain and leukocytes in the process of inflammation (34). Vitamin C reduces peroxides (one of the ROS) and acts as an important antioxidant (35). Honey contains both aqueous and lipophilic antioxidants which enable it to act at different cellular levels as an ideal natural antioxidant (36). This activity decreases the cellular damage caused by free radicals by protecting the antioxidant enzymes and decreasing the oxidative stress, thus decreasing the inflammatory process (37). Schramm and colleagues have concluded that the oral administration of honey can increase the plasma antioxidant level (32). In their study, honey fed at 1.5 gm/kg body weight was found to raise the plasma antioxidant level. Darker color honey with higher water content has more antioxidants. Tualang honey has relatively good antioxidant activity due to the favorable color intensity and phenolic compounds (37)

### 3.4. Anti-viral Properties

There is only one published crossover trial by Al-Waili et al concerning the use of honey in adult patients with recurrent attacks of herpetic lesions (labial and genital) (38). Topical treatment with honey was compared with acyclovir treatment. Honey showed better results with no side effects, over patients using acyclovir who reported itchiness (38). The trial showed that topical honey application was effective in the management of pain and other signs and symptoms of recurrent lesions from genital and labial herpes (38). However, there is a paucity of research regarding the antiviral properties of honey. There have been reports of using medicinal honey in addition to systemic acyclovir in zoster patients with impaired immune systems. This was done with the hope of preventing secondary bacterial infection of the skin as well as speeding up the healing of the herpetic lesions (10). There is enough evidence for clinical use of honey for wound healing. However there is a lack of data in the literature regarding classification of honey’s wound healing properties mechanisms . To make a more informative decision towards using topical honey on different wounds it is important to classify precise mechanisms of action for this effect. Although it is mentioned that honey works on wide spectrum of bacteria, the limitation of this article is that it does not illustrate which species of bacteria are more affected by this treatment. Among the articles reviewed, thirteen published trials lack proper binding, have poor validity and are not free from personal bias. Another eight articles are published by a single researcher. This means that conclusions have to be reviewed with caution. The degree of consistency is considerable in those trials published by the same author; hence, they may have been influenced by personal bias. The size of many trials is questionable (39). More detailed, double blinded randomized controlled studies with sufficient statistical power and minimal personal bias are warranted to investigate the pain relieving, anti-inflammatory and anti-oxidative effects of honey. However, double-blinded studies might not be a possible method for testing wound dressing as honey and its aroma is well recognized (40, 41). Pediatric use cannot be recommended at this stage due to lack of sufficient evidence (41). Similarly, the effects of honey on viruses cannot be advocated due to a paucity of evidence. Although the number of participants treated was small, studies done so far have shown promising results. One positive aspect is the absence of adverse effects in honey treated patients. There are only two adverse effects related to medical honey. One was stinging pain reported by some of the patients (less than 5%), which was resolved by using anaesthetic cream or postponing the treatment to another phase of healing. Secondly, a small number of patients experienced local atopic reactions to medihoney. These patients had an underlying atopic disposition. Therefore, there are no severe systemic reactions to medical honey (10, 42). Factors such as the species of bee, botanical origin and geographical location, as well as processing and storage conditions should be part of the ongoing research (2). A comparison of different types of honey may be an important issue in future research (22).

## 4. Conclusions

Sufficient evidence exists recommending the use of honey in the management of acute wounds and for mild to moderate superficial and partial thickness burns (5). Evidence supporting the use of honey in other areas of clinical practice is needed. Studies revealed that the healing effect of honey could be classified by its antibacterial, antiviral, anti- inflammatory and antioxidant properties of its components. This review should provide practitioners with considerable evidence advocating the use of honey in the medical field. Chronic wound management is an area of perceived confusion, which is why alternative and complementary therapies should be implemented over more conventional treatments. Although some studies exist having tested the efficacy of honey in relation to wound treatment and leg ulcers, more RCTs and systematic reviews of these RCTs could possibly add strength to current evidence. According to the limitation of this study, it is recommended that future researches focus on coverage of the spectrum of anti bacterial effect of honey by using antibiogram.

## References

[1] Zumla A, Lulat A (1989). Honey--a remedy rediscovered.. J R Soc Med..

[2] Moore OA, Smith LA, Campbell F, Seers K, McQuay HJ, Moore RA (2001). Systematic review of the use of honey as a wound dressing.. BMC Complement Altern Med..

[3] Wijesinghe M, Weatherall M, Perrin K, Beasley R (2009). Honey in the treatment of burns: a systematic review and meta-analysis of its efficacy.. N Z Med J..

[4] Khan FR, Ul Abadin Z, Rauf N (2007). Honey: nutritional and medicinal value.. Int J Clin Pract..

[5] Jull AB, Walker N, Deshpande S (2013). Honey as a topical treatment for wounds.. Cochrane Database Syst Rev..

[6] Mohd Zohdi R, Abu Bakar Zakaria Z, Yusof N, Mohamed Mustapha N, Abdullah MN (2012). Gelam (Melaleuca spp.) Honey-Based Hydrogel as Burn Wound Dressing.. Evid Based Complement Alternat Med..

[7] Gethin G, Cowman S (2009). Manuka honey vs. hydrogel--a prospective, open label, multicentre, randomised controlled trial to compare desloughing efficacy and healing outcomes in venous ulcers.. J Clin Nurs..

[8] Lund-Nielsen B, Adamsen L, Kolmos HJ, Rorth M, Tolver A, Gottrup F (2011). The effect of honey-coated bandages compared with silver-coated bandages on treatment of malignant wounds-a randomized study.. Wound Repair Regen..

[9] Yapucu Gunes U, Eser I (2007). Effectiveness of a honey dressing for healing pressure ulcers.. J Wound Ostomy Continence Nurs..

[10] Simon A, Traynor K, Santos K, Blaser G, Bode U, Molan P (2009). Medical honey for wound care--still the 'latest resort'?. Evid Based Complement Alternat Med..

[11] Molan PC (1999). The role of honey in the management of wounds.. J Wound Care..

[12] Al-Waili NS, Salom K, Butler G, Al Ghamdi AA (2011). Honey and microbial infections: a review supporting the use of honey for microbial control.. J Med Food..

[13] Lusby PE, Coombes A, Wilkinson JM (2002). Honey: a potent agent for wound healing?. J Wound Ostomy Continence Nurs..

[14] Molan PC (2006). The evidence supporting the use of honey as a wound dressing.. Int J Low Extrem Wounds..

[15] Molan PC (2002). Re-introducing honey in the management of wounds and ulcers - theory and practice.. Ostomy Wound Manage..

[16] Molan PC, Betts JA (2004). Clinical usage of honey as a wound dressing: an update.. J Wound Care..

[17] Allen KL, Hutchinson G, Molan PC (2000). The potential for using honey to treat wounds infected with MRSA and VRE. First World Wound Healing Congress.

[18] Molan PC, Allen KL (1996). The effect of gamma-irradiation on the antibacterial activity of honey.. J Pharm Pharmacol..

[19] Molan PC (1998). A brief review of the use of honey as a clinical dressing.. Aust J Wound Mange..

[20] Oryan A, Zaker SR (1998). Effects of topical application of honey on cutaneous wound healing in rabbits.. Zentralbl Veterinarmed A..

[21] Aureli P, Franciosa G, Fenicia L (2002). Infant botulism and honey in Europe: a commentary.. Pediatr Infect Dis J..

[22] Molan PC (1992). The antibacterial activity of honey: 1. The nature of the antibacterial activity.. Bee World..

[23] Rushton I (2007). Understanding the role of proteases and pH in wound healing.. Nurs Stand..

[24] Schneider LA, Korber A, Grabbe S, Dissemond J (2007). Influence of pH on wound-healing: a new perspective for wound-therapy?. Arch Dermatol Res..

[25] Tonks AJ, Cooper RA, Jones KP, Blair S, Parton J, Tonks A (2003). Honey stimulates inflammatory cytokine production from monocytes.. Cytokine..

[26] Tonks A, Cooper RA, Price AJ, Molan PC, Jones KP (2001). Stimulation of TNF-alpha release in monocytes by honey.. Cytokine..

[27] Gupta SK, Singh H, Varshney AC, Prakash Prem (1992). Therapeutic efficacy of honey in infected wounds in buffaloes.. Indian J Anim Sci..

[28] Bergman A, Yanai J, Weiss J, Bell D, David MP (1983). Acceleration of wound healing by topical application of honey. An animal model.. Am J Surg..

[29] Tonks AJ, Dudley E, Porter NG, Parton J, Brazier J, Smith EL (2007). A 5.8-kDa component of manuka honey stimulates immune cells via TLR4.. J Leukoc Biol..

[30] Al-Waili N, Salom K, Al-Ghamdi AA (2011). Honey for wound healing, ulcers, and burns; data supporting its use in clinical practice.. Sci World J..

[31] Sherlock O, Dolan A, Athman R, Power A, Gethin G, Cowman S (2010). Comparison of the antimicrobial activity of Ulmo honey from Chile and Manuka honey against methicillin-resistant Staphylococcus aureus, Escherichia coli and Pseudomonas aeruginosa.. BMC Complement Altern Med..

[32] Schramm DD, Karim M, Schrader HR, Holt RR, Cardetti M, Keen CL (2003). Honey with high levels of antioxidants can provide protection to healthy human subjects.. J Agric Food Chem..

[33] van den Berg AJ, van den Worm E, van Ufford HC, Halkes SB, Hoekstra MJ, Beukelman CJ (2008). An in vitro examination of the antioxidant and anti-inflammatory properties of buckwheat honey.. J Wound Care..

[34] Bashkaran K, Zunaina E, Bakiah S, Sulaiman SA, Sirajudeen K, Naik V (2011). Anti-inflammatory and antioxidant effects of Tualang honey in alkali injury on the eyes of rabbits: experimental animal study.. BMC Complement Altern Med..

[35] Park DV (1999). Antioxidants in human health and tissue: Nutritional antioxidantsand disease prevention: Mechanism of action..

[36] Aljadi AM, Kamaruddin MY (2004). Evaluation of the phenolic contents and antioxidant capacities of two Malaysian floral honeys.. Food Chem..

[37] Erejuwa OO, Sulaiman SA, Wahab MS, Sirajudeen KN, Salleh MS, Gurtu S (2010). Antioxidant protection of Malaysian tualang honey in pancreas of normal and streptozotocin-induced diabetic rats.. Ann Endocrinol (Paris)..

[38] Al-Waili NS (2004). Topical honey application vs. acyclovir for the treatment of recurrent herpes simplex lesions.. Med Sci Monit..

[39] Moore RA, Gavaghan D, Tramer MR, Collins SL, McQuay HJ (1998). Size is everything--large amounts of information are needed to overcome random effects in estimating direction and magnitude of treatment effects.. Pain..

[40] Shmueli A, Shuval J (2007). Are users of complementary and alternative medicine sicker than non-users?. Evid Based Complement Alternat Med..

[41] Bell SG (2007). The therapeutic use of honey.. Neonatal Netw..

[42] Al-Waili NS, Saloom KY (1999). Effects of topical honey on post-operative wound infections due to gram positive and gram negative bacteria following caesarean sections and hysterectomies.. Eur J Med Res..

